# New Delhi metallo-β-lactamase-1 among *Escherichia coli* strains isolated from leukemia patients in Iran: two case reports

**DOI:** 10.1186/s13256-021-03160-2

**Published:** 2021-11-25

**Authors:** Mahdane Roshani, Alireza Goodarzi, Sanaz Dehbashi, Farhad Afrasiabi, Hossein Goudarzi, Ali Hashemi, Mohammad Reza Arabestani

**Affiliations:** 1 Department of Microbiology, Faculty of Medicine, Nutrition Research Center, Hamadan University of Medical Sciences, Shahid Fahmideh Street, Park Mardome, Hamadan, IR Iran; 2 Department of Medical Laboratory Sciences, School of Paramedicine, Hamadan University of Medical Sciences, Shahid Fahmideh Street, Park Mardome, Hamadan, IR Iran; 3 Department of Microbiology, Shahid Beheshti University of Medical Sciences, Shahid Shahriari Square, Daneshjou Boulevard, Shahid Chamran Highway, 1983969411 Tehran, Iran

**Keywords:** *bla*_NDM-1_, *Escherichia coli*, *Klebsiella pneumoniae*, Leukemia

## Abstract

**Background:**

*Escherichia coli* has appeared as an important opportunistic pathogen responsible for nosocomial infections in patients with immunodeficiency, particularly in leukemia patients. New Delhi metallo-beta-lactamase is an enzyme originally found in Enterobacteriaceae.

**Case presentation:**

In this study, 80 isolates of *Escherichia coli* and *Klebsiella pneumoniae* were collected over the course of 2 years from two medical centers in Tehran, Iran. Production of carbapenemase was detected in the isolates using modified Hodge test. New Delhi metallo-beta-lactamase-1 genes were detected by polymerase chain reaction amplification with specific primers. Two New Delhi metallo-beta-lactamase-1-producing *Escherichia coli* strains were isolated from two Iranian patients with leukemia. These two patients were 6 and 15 years old, one female and the other male, from two oncology centers in Iran. The isolates were resistant to carbapenems (imipenem, meropenem), and two isolates were positive for carbapenemase production by modified Hodge test.

**Conclusions:**

The emergence of New Delhi metallo-beta-lactamase-1-producing *Escherichia coli* is a threat for leukemia patients in oncology and hematology departments. We conclude that the incidence of multidrug resistant pathogens has increased among patients with leukemia and is life threatening.

## Introduction

The globally increasing prevalence of New Delhi metallo-β-lactamase-1 (NDM-1)-producing Enterobacteriaceae is a concerning phenomenon in immunocompromised patients. Previous results indicated that *bla*_NDM-1_ gene can be carried on incompatibility group N (IncN) plasmids of different sizes along with other resistance factors. *bla*_NDM-1_ can confer resistance to almost all the β-lactams. Thus, bacteria carrying NDM-1 gene are considered to be resistant to all antibiotic classes except colistin and ciprofloxacin. The gene was scarcely integrated into the chromosome. The sequencing of this gene suggests a new enzyme, unrelated to hitherto known metallo β-lactamases (MBLs). The most similar known type is Verona integron-encoded metallo-β-lactamase (VIM-1/VIM-2), sharing 32.4% resemblance [1]. Immunosuppressive, sepsis, and radiation therapy can be the differential diagnosis of exotic infection [2–5].

In a study conducted in China, the presence of this gene was first reported in a patient with leukemia, but it has mainly been reported in patients with neutropenia. Our study is the first report from Iran. The purpose of this study was to investigate the existence of NDM-1 gene as a risk factor for life-threatening infection in patients with leukemia.

## Case presentation

In this study, 80 isolates of *Escherichia coli* and *Klebsiella pneumoniae* were collected from two medical centers in Tehran, Iran: the Hematology-Oncology Research Center, Dr. Shariati Hospital, and the Mahak Pediatric Oncology Center, between 2014 and 2015.

The bacteria were isolated and stored in Hamadan University of Medical Sciences. All the clinical specimens were quickly sent to the laboratory and analyzed for confirmatory test. Isolates were identified using standard microbiological and biochemical procedures [6]. In our previous study, antibiotic susceptibility of the isolates was tested by Kirby–Bauer disk diffusion method for imipenem, ceftazidime, ceftriaxone, cefotaxime, ciprofloxacin, levofloxacin, amikacin, ampicillin, and gentamicin (all from Mast, UK). Moreover, the combination disk method was employed to detect extended-spectrum β-lactamase (ESBL)-producing isolates, while the minimum inhibitory concentrations (MICs) of selected antimicrobials were determined by the broth microdilution method. The results were interpreted according to the Clinical Laboratory Standards Institute (CLSI) guidelines [7]. The carbapenem-resistant strains were investigated for carbapenemase production by modified Hodge test (MHT) according to the CLSI guidelines, in which *E. coli* ATCC25922 was used as the positive control [8]. Furthermore, the combined disk diffusion method was applied for *bla*_MBL_ detection using two IPM (10 µg) disks and ethylenediaminetetraacetic acid (EDTA) 0.5 M solution[9]. For diagnosis of the NDM1, polymerase chain reaction (PCR) was performed on DNA extracted by boiling, using NDM-F-specific primers: CAACTGGATCAAGCAGGAGA, NDM-R TCGATCCCAACGGTGATATT (Bioneer Company, Korea) [10].

The sequencing of amplicons in both directions was carried out by the Bioneer Company (Daejeon, South Korea). The data were analyzed using FinchTV software (Geospiza, USA), and the sequences were then confirmed using the National Center for Biotechnology Information (NCBI) website (http://www.ncbi.nlm.nih.gov/ BLAST). A total of 56 *E. coli* and 24 *K. pneumoniae* isolates were obtained from urine, blood, sputum, wound, and vagina. Among the 80 isolates, 52 (63%) strains were ESBL producers, followed by 5 (6.25%) metallo β-lactamase (MBL) producers. According to our antimicrobial susceptibility test on *E. coli* and *K. pneumoniae* isolates, eight (10%) of *E. coli* isolates were imipenem-resistant. Out of 80 isolates, 8 (10%) isolates were found to be carbapenem-resistant. The major MBL and carbapenem-resistant species were *E. coli*. The first NDM-1-containing isolate was obtained from a 26-year-old subject diagnosed with acute leukemia. The second was obtained from a 2-year-old child with acute leukemia. These two NDM-1-positive *E. coli* isolates (labeled as E1–E2) were recovered from the urine and blood samples of two different hospitalized patients. Both isolates were positive for the modified Hodge test and MBL producers (Tables 1 and 2).

**Table 1:** Antimicrobial resistance profile of two NDM-1-producing *E. coli* isolates from two patients with leukemia

Phenotypic tests for carbapenemase		Minimum inhibitory concentration (μg/mL) for antibiotics	
Strains	MHT	IMP	MEM
E1	+	4	8
E2	+	16	32

**Table 2:** Distribution of cefotaxime-resistant (CTX-M) group, *qnr* genes, *TEM* gene, IS903, IS26, and ISEcp1 elements in NDM-1-producing *E. coli* isolated from leukemia patients

	CTXM1	CTXM2	CTXM8	CTXM9	CTXM25	ISECP1	IS26	IS903	*qnrA*	*qnrB*	*qnrS*	*TEM*
E1	+	−	−	+	-	+	+	+	+	+	−	+
E2	+	−	−	−	−	+	+	−	−	−	−	+

## Discussion

The present study reports NDM-1-producing *E. coli* strain from the bloodstream and infected urinary tract of two patients. PCR results confirmed that the NDM-1-producing *E. coli* harbored *qnrA*, *qnrB*, *qnrS* and *bla*_CTXM-1_,_CTX-M2_,_CTX-M8_,_CTX-M9_,_CTX-M25_, and the insertion sequence of ISECP1,IS26,IS903. The two NDM-1-producing *E. coli* isolates did not contain *qnrS* genes, but they carried *bla*_TEM_ gene.

Eyvazi *et al.* recently reported *bla*_NDM-1_-producing *E. coli* in Iran for the first time [11]. Shahcheraghi *et al.* detected the first *bla*_NDM-1_-producing *K. pneumonia* in Iran as well [12].

In October 2011, Laurent Poirel *et al.* reported *bla*_NDM-1_-producing *K. pneumoniae* and *E. coli*. in a 16-year-old male patient admitted to the hematology unit of a hospital in Istanbul, Turkey [13].

In a study in 2010 on a patient transferred from Iraq to France, NDM-1-producing *K. pneumoniae* was also reported [14].

Reports have also declared the existence of *bla*_NDM-1_-producing Enterobacteriaceae in Pakistan and Afghanistan [15, 16]. The *bla*_NDM-1_-producing bacteria could also be found in environmental samples and drinking water [17]. In our investigation, PCR confirmed that the NDM-1-producing *E. coli* harbored quinolone resistance gene B (*qnrB*) and *bla*_CTXM-9_. This result was similar to the other reports on strains carrying *bla*_NDM-1_ that also harbored other β-lactamase genes [18].

An NDM-1-producing *E. coli* strain was detected in the bloodstream of a patient in this study that exhibited high resistance to all tested β-lactam antibiotics, which can be attributed to the production of *bla*_NDM-1_ and other resistant genes.

Bahramian *et al.* published the first report about metallo-β-lactamase-6 (NDM-6) among *K. pneumoniae* in New Delhi. ST147 strains were also isolated from dialysis patients in Iran [19]. Firoozeh *et al.* reported 20 (11.1%) *K. pneumoniae* isolates harboring *bla*_NDM-1_ gene (20).

We inferred that it is important to evaluate the health condition of immunocompromised patients, especially those with leukemia. Moreover, patients receiving chemotherapy may develop bloodstream infections. Bacterial infections can result in significant morbidity and mortality due to the development of febrile neutropenia and bacteremia.

## Conclusion

This is the first report on the *bla*_NDM-1_-producing *E. coli* strains isolated from leukemia patients in Iran. NDM-1-producing *E. coli* also harbored genes encoding cefotaxime-resistant (CTX-M) group, TEM, quinolone resistance (QNR), and insertion sequence of resistance enzymes. The coincidence of *NDM-1* with other antibiotic-resistance genes may further limit the treatment options and makes infection control procedures more challenging among leukemia patients. Our results show the emergence of *bla*_NDM-1_ as an alarm to our health services, particularly among immunocompromised patients.

## Data Availability

The datasets used and/or analyzed during the current study are available from the corresponding author on reasonable request.

## Abbreviations

NDM-1: New Delhi metallo-β-lactamase-1
ESBL: Extended spectrum β-lactamase
MIC: Minimum inhibitory concentration
MBL: Metallo-β-lactamase
VIM: Verona integron-encoded metallo-β-lactamase
Qnr: Quinolone resistance
bla: Beta lactamase
